# Influence of dental prophylaxis procedures on the tooth veneer interface in resin-based composite and polymer-infiltrated ceramic veneer restorations: an in vitro study

**DOI:** 10.1007/s00784-022-04816-z

**Published:** 2022-12-11

**Authors:** Lena Unterschütz, Florian Fuchs, Laura-Antonia Mayer, Andreas Koenig, Nadia Challakh, Ellen Schulz-Kornas, Dirk Ziebolz, Sebastian Hahnel

**Affiliations:** 1 Department of Prosthetic Dentistry and Dental Material Science, Leipzig University, Liebigstraße 12, 04103 Leipzig, Germany; 2 Department of Cariology, Endodontology and Periodontology, Leipzig University, Liebigstraße 12, 04103 Leipzig, Germany; 3 Department of Prosthetic Dentistry, UKR University Hospital Regensburg, Franz-Josef-Strauß-Allee 11, 93053 Regensburg, Germany

**Keywords:** Surface analysis, Interface, Veneer, Air polishing, Ultrasonic scaling

## Abstract

**Objectives:**

The aim of this study was to investigate the influence of dental prophylaxis cleaning procedures and artificial aging on veneers in human teeth. The external marginal and internal tooth veneer as well as the restoration surfaces were examined.

**Material and methods:**

Thirty-two extracted premolars were restored with resin-based composite (RBC) and polymer-infiltrated ceramic network (PICN) veneers. Artificial aging by alternating thermocycling and subsequent prophylaxis procedure (glycine-based powder air polishing or ultrasonic scaling) was conducted for five consecutive cycles. The external marginal interface was examined by height profile measurements and the internal interface was investigated using micro X-ray computed tomography. In addition, the surface texture of the veneer surface was analyzed using confocal laser scanning microscopy.

**Results:**

The application of both prophylaxis procedures resulted in a deepening of the marginal interface (10 µm ± 8 µm) for materials. Furthermore, the internal interface of PICN restorations showed marginal gaps after both treatments and artificial aging (16 µm ± 3 µm). In contrast to the RBC specimens, a significant increase in surface roughness was identified for PICN veneers after ultrasonic scaling.

**Conclusions:**

The marginal and internal interface regions in veneers fabricated from PICN and RBC were affected by prophylaxis procedures. Furthermore, it may result in increased veneer surface roughness, especially in PICN and after ultrasonic scaling, which might affect bioadhesion and longevity.

**Clinical relevance:**

After dental prophylaxis procedures, examination of the marginal and the internal interface as well as the veneer surface provides a precise insight into damage mechanisms and offers an assessment of longevity.

**Supplementary Information:**

The online version contains supplementary material available at 10.1007/s00784-022-04816-z.

## Introduction

The prevalence of noncarious defects, such as erosion, abrasion, attrition, and abfraction, is steadily increasing in the European population [1, 2]. These diseases regularly require minimally invasive dental treatment, which has become possible with the continuous improvement of tooth-colored dental materials. These materials can be adhesively luted to dental tissues and allow defect-oriented tooth preparation as well as the accomplishment of esthetic outcomes [3, 4]. Veneer restorations particularly respond to these demands. Originally introduced for the esthetic remodeling of the labial surfaces of anterior teeth, the application of veneers has also become popular as a minimally invasive means to restore the occlusal surfaces in eroded or worn posterior teeth [5, 6]. With the continuous development of computer-aided design/computer-aided manufacturing (CAD/CAM) technology, it is possible to use this type of treatment to meet the current conditions and demands in the dental field [7].

Veneers can be fabricated from a variety of materials, including hybrid materials, such as resin-based composites (RBC) and polymer-infiltrated ceramic network materials (PICN) [7, 8]. Although silicate ceramics have been the material of choice for a long time, RBCs and PICNs require no firing and feature the advantage of easier laboratory and clinical processing, which finally reduces the time required for fabrication and adjustment of the restoration [9]. Moreover, these hybrid materials feature higher fracture resistance, lower brittleness, and a tooth-like flexural modulus and strength, and intraoral repairs can be more easily performed compared with ceramic restorations [9–12]. Some researchers also highlight that restorations fabricated from these materials produce less stress on antagonist teeth [13–15]. As a result of their improved marginal stability, the margins of PICN can be thinner compared with those of silicate ceramics, which facilitates the minimally invasive character of veneer restorations [7].

The relevance of dental prophylaxis procedures has been continuously increasing over the last decades [16], which might be explained by a growing consciousness in the general population regarding health issues. Although the frequency of preventive measures depends on the individual requirements of each patient, a minimum of one prophylactic dental treatment session per year is often recommended. Professional tooth cleaning can be performed with a variety of instruments, including ultrasonic scaling and powder polishing devices. Several studies have shown that the use of these instruments can affect the surface properties of natural tooth tissue by increasing roughness [17, 18], and RBC and PICN can be used to finally foster the accumulation of biofilms [19–21]. In addition, it has also been reported that ultrasonic scaling may also impair the adhesive bonding between tooth tissues and light-cured resin-based composite restorations [22]. While existing studies focus either on the effect of prophylactic treatment on the surface of the restorative material [19, 23], the internal adhesive interface [24], or accelerated aging [25], the influence of a combined approach has not yet been analyzed.

Scientific data on the long-term performance of veneer restorations are scarce, yet current evidence highlights that veneer restorations in anterior teeth feature 10-year survival rates ranging approximately 75.0% for veneers fabricated from RBCs [26]. Reasons for failure of veneer restorations include microleakage, debonding, fractures, and individual patient-related factors. It has been underlined that marginal discoloration and marginal discrepancies are regularly observed issues as well as increased surface roughness [26, 27]. Thus, it might be possible that treatment measures employed for professional tooth cleaning negatively influence the interface or the surface of the restoration itself.

Against this background, this study investigates the influence of two common dental treatment procedures for professional tooth cleaning on the marginal and internal tooth veneer interface of RBC and PICN veneers with a time-accelerated artificial aging approach. In addition, the influence of the treatment on the surface texture of the veneers is analyzed. It was hypothesized that there is no difference between the two prophylactic treatments in terms of their individual influence on (i) the marginal or (ii) the internal tooth veneer interface as well as (iii) the surface texture of the veneer restoration.

## Material and methods

### Specimen preparation

Thirty-two extracted human premolars evenly distributed from the maxilla and mandible with intact and caries-free labial surfaces were used. Premolars were stored in 0.5% chloramine-T solution at 4 °C until use. The following exclusion criteria were applied: endodontic treatment, fractures, and direct and indirect restorations. All premolars were prepared by two experienced dentists (L.U., L.-A.M.) in accordance with the conventional guidelines for veneer restorations fabricated from RBC and PICN. An average vestibular reduction of 0.4 mm was set using a depth marking instrument (“PrepMarker”), and the procedure was performed using fine and extra fine diamond burs (head shapes: football, flame and round end tapered, see supplemental material Table S1). The transition zone ranging from the buccal to the coronal and occlusal area was reduced by approximately 1.5 mm using the incisal reduction approach commonly performed for anterior teeth. The selected preparation design is supposed to resemble the palatal chamfer or incisal overlap. Rounded shoulder margins were produced all over the entire preparation. Digital impressions were taken from the preparation molds (Primescan AC 172 with Cerec SW 5 Software Version 5.1.0.190461, Dentsply Sirona, York, Pennsylvania, USA). The restorations were digitally constructed using the “InLab 19.0” design software (Dentsply Sirona, York, PA, USA). The selected thickness of the veneers for spacer application was set to 80 µm [28]. Using an “InLab MC XL” milling machine (Dentsply Sirona, York, PA, USA), 16 veneers were fabricated from each CAD/CAM resin-based composite RBC (Grandio Blocs A2 HT, Voco GmbH, Cuxhaven, Germany) and polymer-infiltrated ceramic network material PICN (Enamic 2M2 HT, Vita Zahnfabrik, Bad Säckingen, Germany). Protruding milling edges were manually removed using an extrafine diamond bur with continuous water cooling. PICN veneers were etched with 5% hydrofluoric acid for 60 s [29]; RBC veneers were sandblasted with aluminum oxide (50 µm, 1.5–2.0 bar, 60 s) [30]. For both types of veneers, the application of “Ceramic Primer Plus” was followed by etching the teeth with the “K-Etchant” for 10 s, rinsing, and drying for 20 s after the application of “Tooth Primer” (all materials: Panavia V5 Professional Kit). Afterwards, the veneers were adhesively luted using a resin-based dental cement (Panavia V5, Color A2) [31] and polished with fine diamond burs and ceramic or composite polishers in accordance with the protocols published by Matzinger et al. (2019) [32]. For reproducible alignment during analysis, the roots of the teeth were embedded in fast-curing 3-component resin (Technovit 4000). The materials used for preparation of the teeth are summarized in the supplemental material (Table S1).

### Accelerated aging and prophylaxis procedures

To investigate the long-term outcomes of prophylaxis procedures on the tooth veneer interface, the specimens were repeatedly exposed to alternating thermal stress followed by prophylactic treatment for five consecutive cycles. Therefore, the premolars were split into four groups with eight specimens each:

- RBC or PICN treated with ultrasonic scaling.
- RBC or PICN treated with glycine-based powder air polishing.

All 32 restored teeth were initially subjected to thermocycling (5/55 °C; 5000 cycles) with a holding time of 30 s and a 5 s pause between the two water baths (Thermocycler THE, SD Mechatronics, Feldkirchen-Westerham, Germany). A total of five aging runs were alternated with prophylactic treatment in each individual group. The thermal stress is based on the widely used and recommended ISO 11405 for better comparability with other studies [33, 34]. Since this is a temperature load (5000 cycles, 5/55 °C) with a higher thermal stress but a half the number of cycles than the temperature protocol recommended by Gale and Darvell (1999) for simulating a year in clinical practice (10,000 cycles, 35/15/35/45 °C), the authors also assumed an approximate simulation of a year (and a total of five runs for 5 years) [35].

**Fig. 1:**
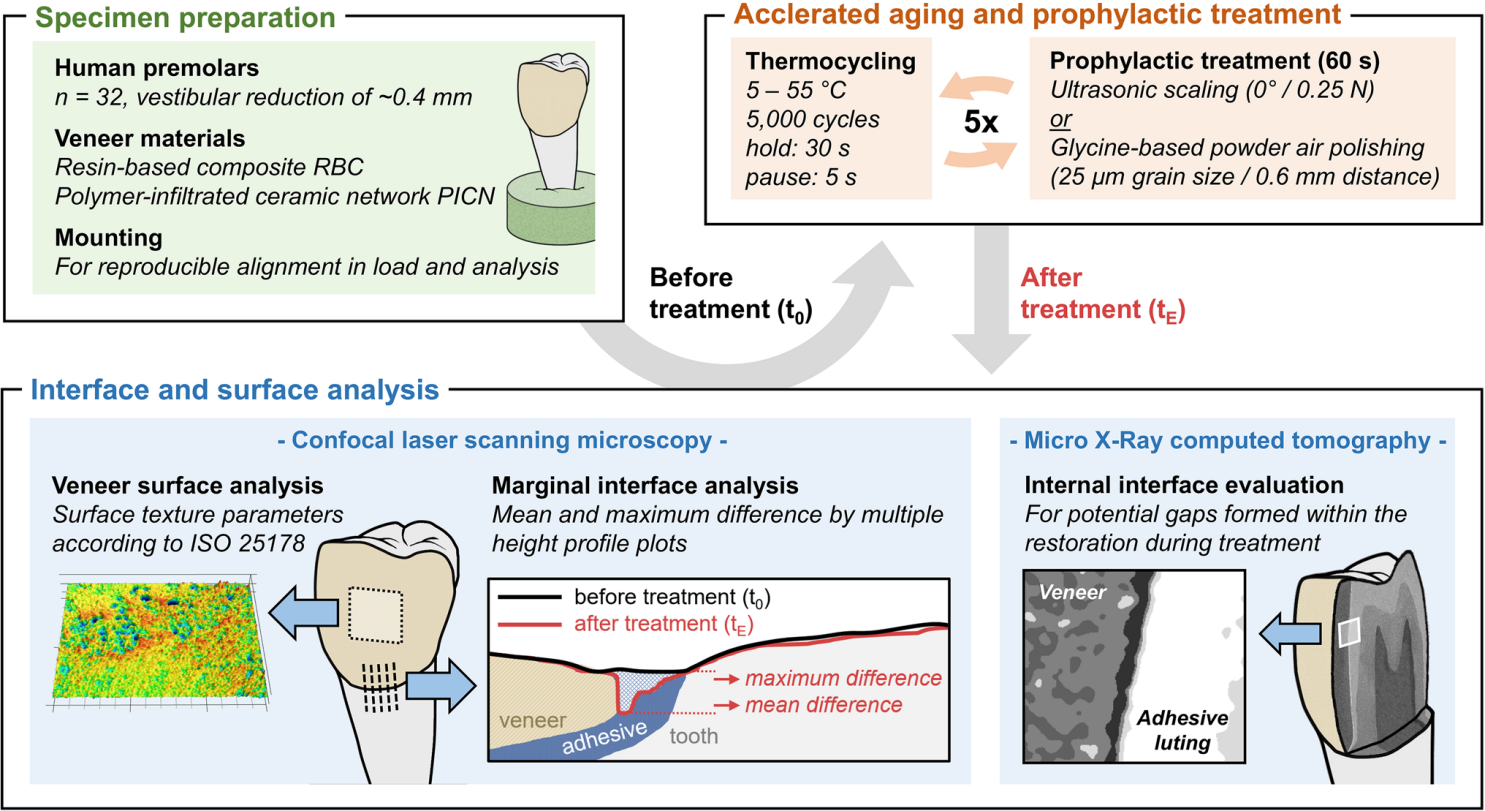
Overview of sample preparation. Accelerated aging including prophylactic treatment and analytical procedures for analysis of the marginal and internal interface (transition area between veneer, adhesive, and tooth structure) as well as the veneer surface

Subsequently, the specimens were randomly allotted to two subgroups. The vestibular surface of each specimen was treated for 60 s with either a magnetostrictive ultrasonic scaler (Cavitron FSI Slimline 30 K, DentsplySirona, Charlotte, NC, USA) with a straight 10S attachment or an air polishing regime (Perio Powder and Airflow Handy 3.0 Perio, grain size: 25 µm, EMS Dental, Nyon, Switzerland) (Fig. 1). To standardize the treatments, a specially developed in vitro laboratory setup was used to ensure constant powder blasting with a spacing of 6 mm and an angulation of 30–60° blasting from the coronal direction [36]. The blasting process was performed in a circular motion. Regarding the application of the ultrasonic scaler, the embedded specimens were held in hand, and the surface was processed at an angle of 0° and a manually adjusted contact pressure of approximately 0.25 N [37]. The pressure was calibrated manually with the aid of a precision scale (PCB 3500–2, Kern, Ballingen, Germany) prior to each application.

### Height profile analysis of the marginal interface and surface texture

For the evaluation of changes in the marginal interface region, the surface morphology, and texture of the veneers, a confocal laser scanning microscope (VK-X1000/X1050, Keyence, Osaka, Japan) was used with 20 × magnification (CF IC EPI Plan 20 × ; *N* = 0.46; WD = 3.1 mm, field of view 675 × 506 µm), a red laser (*λ* = 661 nm), and a resolution of 2048 × 1536 pixels. Measurements were obtained using “VK Viewer 1.1.2.174” software (Keyence, Osaka, Japan) at two times prior to (*t*_0_) and after (*t*_*E*_) completing the treatments. Data analysis was performed using the software “MultiFileAnalyzer” 2.1.3.89 (Keyence, Osaka, Japan).

For the analysis of the marginal interface (i), coronal to apical height profiles were analyzed based on the surface measurement of the interface region. The surface measurements were subjected to shape correction by an F-Filter (0.25 mm) following ISO 25178, resulting in S-F surfaces that have been further analyzed. Each (*t*_0_) and (*t*_*E*_) measurement was manually positioned and aligned using a software-based automatic position adjustment (via MultiFileAnalyzer). In addition, a manual examination verifying the conformity of previously defined surface sites in the region of the tooth and veneer was conducted. Within each interface measurement, 15 height profile lines perpendicular to the adhesive interface with a length of 400-µm and 5-µm intervals were analyzed. Finally, the average (DiffMean) and maximum (DiffMax) difference between the (*t*_0_) and (*t*_*E*_) profile lines within the area of the tooth veneer interface were measured and averaged for all 15 measurements (Fig. 1).

For the determination of surface texture (iii), measurements of the veneer at a central position were quantified according to ISO 25178 (Fig. 1). Short-scale filtering was applied to measure the surface roughness of the veneers on the S-L surface: S-filter, 2 µm; F-filter, 0.25 mm; L-filter, 0.05 mm; filter type, double Gaussian. Evaluation of surface morphology on the long-scale (“waviness”) was performed using the S-F surface with the following filter settings: S-filter, 50 µm; F-filter, 0.25 mm; filter type, double Gaussian [38]. Both surfaces were quantified using four selected height parameters, including arithmetic mean height (*Sa*, µm), root-mean-square height of the surface (*Sq*, µm), skewness (*Ssk*, no unit), kurtosis (*Sku*, no unit); three functional parameters, including core roughness depth (*Sk*, µm), reduced summit height (*Spk*, µm), reduced valley depth (*Svk*, µm); and one hybrid parameter, namely, developed interfacial area ratio (*Sdr*, converted into %) following previous protocols [23, 39, 40].

For an assessment of changes in the esthetic appearance of the restorations, all specimens were additionally photographed before and after exposure using macrophotography as reported by Fuchs et al. (2020) [41]. Representative comparison images are included in the supplemental material (Figure S1, Figure S2).

### Microstructure analysis of the internal interface

For the nondestructive analysis of the internal interface (ii) and its bonding to tooth tissues and restoration, three samples per material and prophylaxis procedure were investigated using micro X-ray computed tomography (µXCT, FhG-IKTS-MD, Dresden, Germany) (Fig. 1). An X-ray microfocus tube in transmission mode (FXE 225.99, YXLON International GmbH, Hamburg, Germany) with a focal spot of 0.6 µm, tungsten target, 0.1-mm copper filter, and a constant beam energy (180 kV/150 µA) with a 2D-detector (2048 × 2048 pitches, CsI; PerkinElmer Inc., Waltham, MA, USA) was employed. The samples were rotated in a full circle of 360° with a step size of 45°. A resolution of 7 µm per voxel could be achieved within the settings. The single images were reconstructed with the software “Volex 6.2” (FhG, Dresden, Germany), processed with “ImageJ 1.47 v” (National Institutes of Health, Bethesda, MD, USA), and referenced according to Koenig (2020) [42]. The 3D datasets gathered at (*t*_0_) and (*t*_*E*_) were compared. Marginal gaps identified in the area at the veneer-composite interface (Fig. 3) were manually measured using three different sectional views at a distance of 50 slices (approx. 350 µm) with 10 manually set distance measuring lines each.

### Statistical analysis

Both data for surface profiles and surface texture parameters were tested for normal distribution using the Shapiro–Wilk test. Differences in surface parameters at time (*t*_0_) and (*t*_*E*_) were evaluated by *t*-tests (no change in parameters due to application of prophylaxis procedure and accelerated aging). Comparison of surface profiles by material and treatment was performed by Welch-ANOVA using Levene's test, considering equality of variances. The level of significance (*α*) was set to 0.05. The length measurements of the gap in the region of the internal interface by CT analysis were averaged as the mean value. IBM SPSS Statistics 27.0.1.0 software was used for the statistical tests.

## Results

### Survival rate

Thirty-one of 32 veneers (96.9%) survived all five test cycles (*t*_0_)-(*t*_*E*_). One RBC veneer restoration in the air polishing group failed adhesively due to debonding in the area between the veneer and the adhesive cement after the second round of thermocycling and prophylaxis procedures.

### Marginal interface

**Fig. 2:**
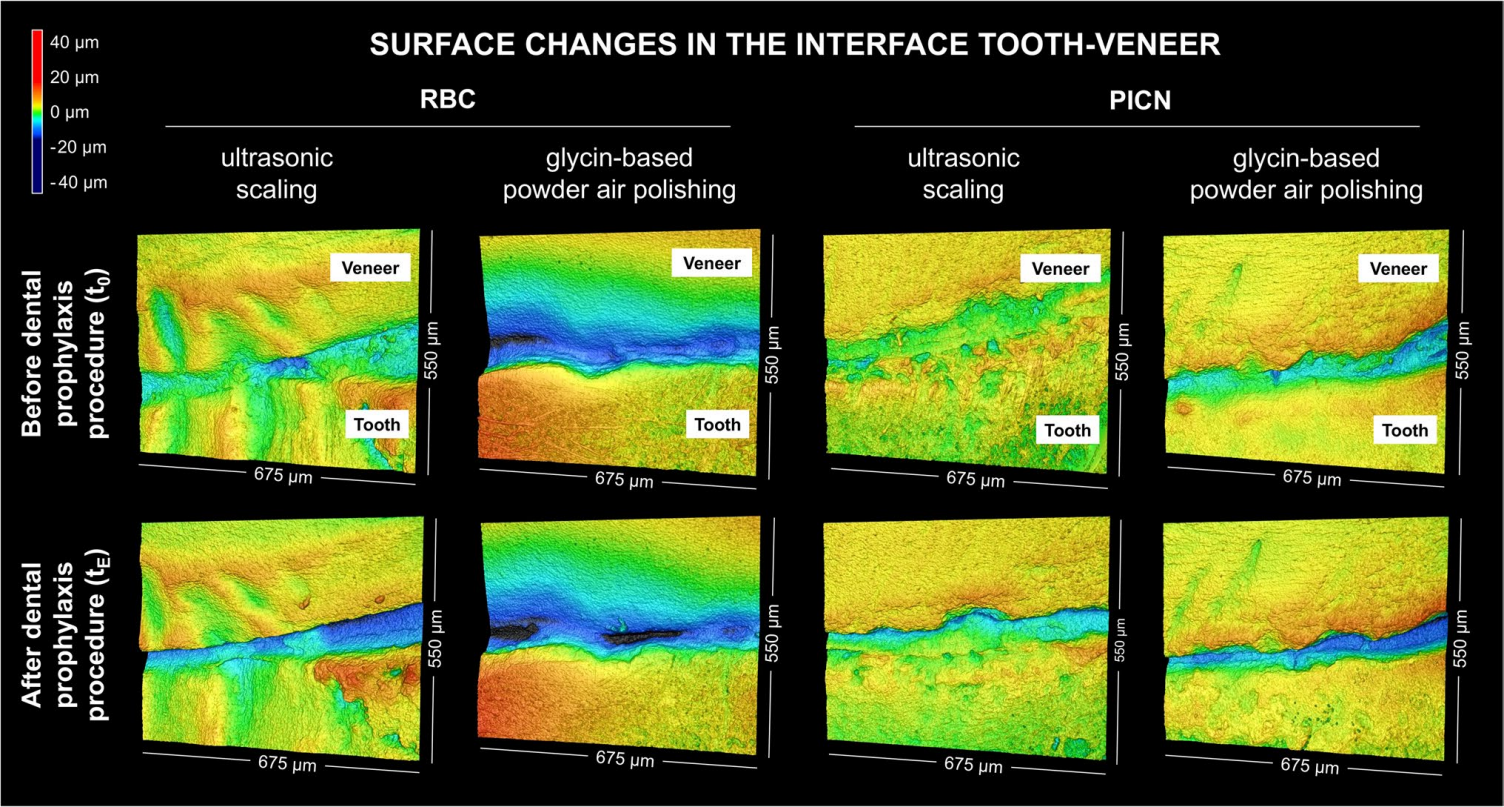
Visual appearance of the interface tooth veneer of resin-based composite (RBC) and polymer-infiltrated ceramic network material (PICN) veneer surfaces (3D models of S-F surfaces) prior to and after exposure to dental prophylaxis procedure

In general, for all groups, the visual impression of the rendered 3D models of the tooth veneer interface indicated an increase in depth in the area of the luting composite after exposure to ultrasonic scaling or air polishing, which could be visualized by CLSM measurements (Fig. 2). This observation was also quantitatively verified by the results of DiffMean 4.79 µm (3.69) and DiffMax 10.39 µm (8.18) (Table 1). No significant differences in DiffMean or DiffMax were identified between the various materials and treatments (*p* = 0.211 and *p* = 0.135, respectively).

**Table 1:** Measured values and standard deviation (SD) for mean (DiffMean) and maximum (DiffMax) differences between the plot profiles across the tooth veneer interface before and after loading of the specimens

Differences between surface plot profiles *t*_0_ and *t*_*E*_	RBC		PICN	
	Ultrasonic scaling	Air polishing	Ultrasonic scaling	Air polishing
DiffMean (SD)/µm	3.10 (1.30)	6.36 (4.96)	4.15 (3.42)	5.74 (4.08)
Mean of all samples (SD)/µm	4.79 (3.69)			
DiffMax (SD)/µm	6.40 (2.37)	16.32 (11.96)	9.36 (8.00)	10.23 (6.31)
Mean of all samples (SD)/µm	10.39 (8.18)			

### Internal interface

**Fig. 3:**
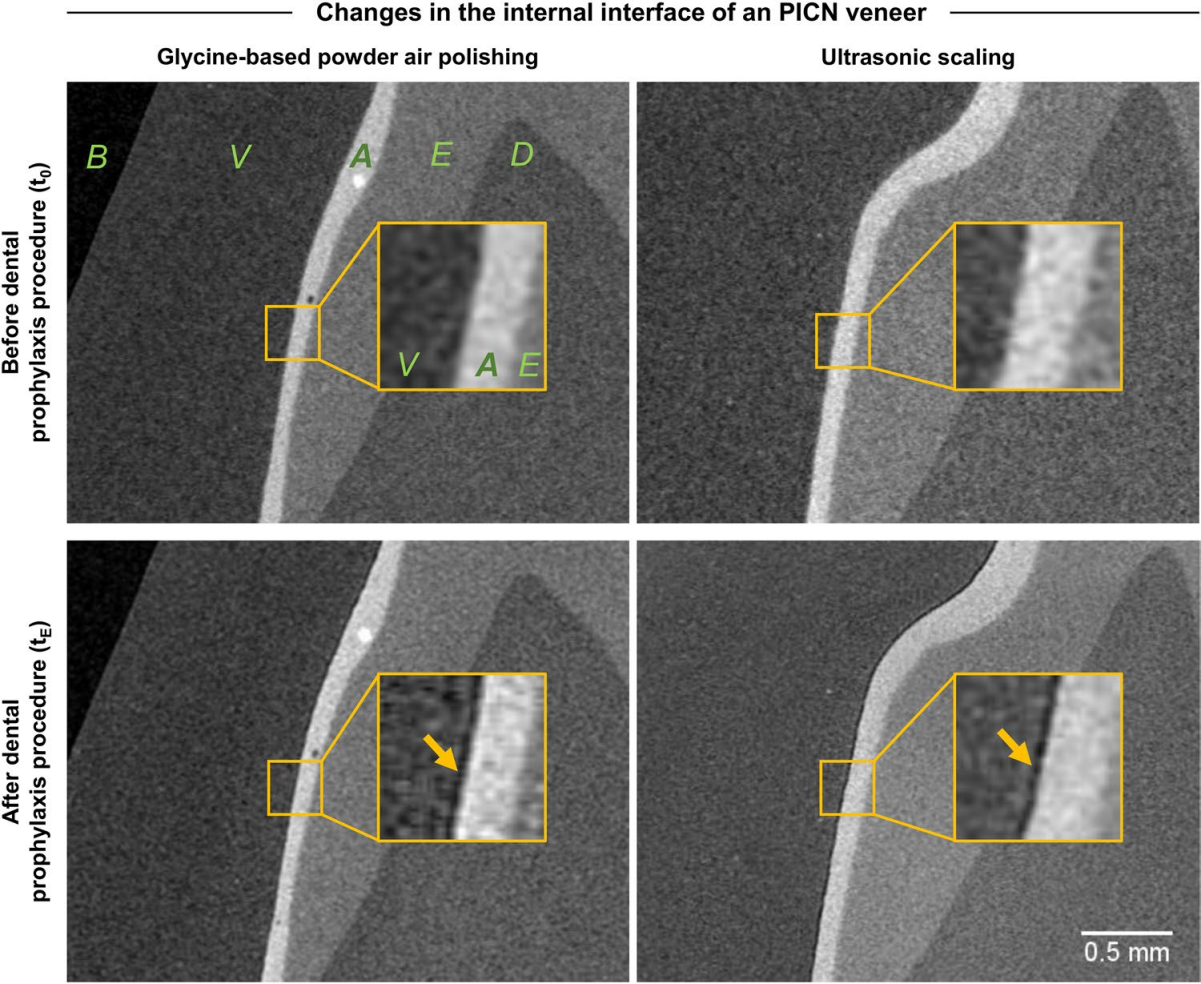
µXCT sectional images of a specimen with veneer (V), adhesive layer (A), enamel (E), dentin (D), and background (B) with an enlarged region of interest (ROI) of the µXCT datasets of a polymer-infiltrated ceramic network material (PICN) veneer before (*t*_0_) and after (*t*_E_) treatment; left: treated with air polishing, right: treated with ultrasonic scaling; yellow box: a gap (yellow arrow) formed between veneer and adhesive layer in both (*t*_E_) specimens

Three randomized specimens from each group, material, and prophylactic treatment were analyzed with µXCT. To show the resulting damage at the internal interface, only the selection of specimens with clear gaps or visible expansion in the adhesive layer are displayed in this study. The 3D examination revealed differences within three particular specimens in the interface between (*t*_0_) and (*t*_*E*_). In two PICN specimens, including one treated with ultrasonic scaler and one with glycine air polish, a gap was identified between the veneer and the adhesive layer. The interface showed a gap between the veneer and the resin-based dental cement of 15.9 µm (2.7) for glycine-based powder air polishing and 16.8 µm (2.4) for ultrasonic scaling (Fig. 3). In the RBC group, no such gaps were discovered in this area.

**Fig. 4:**
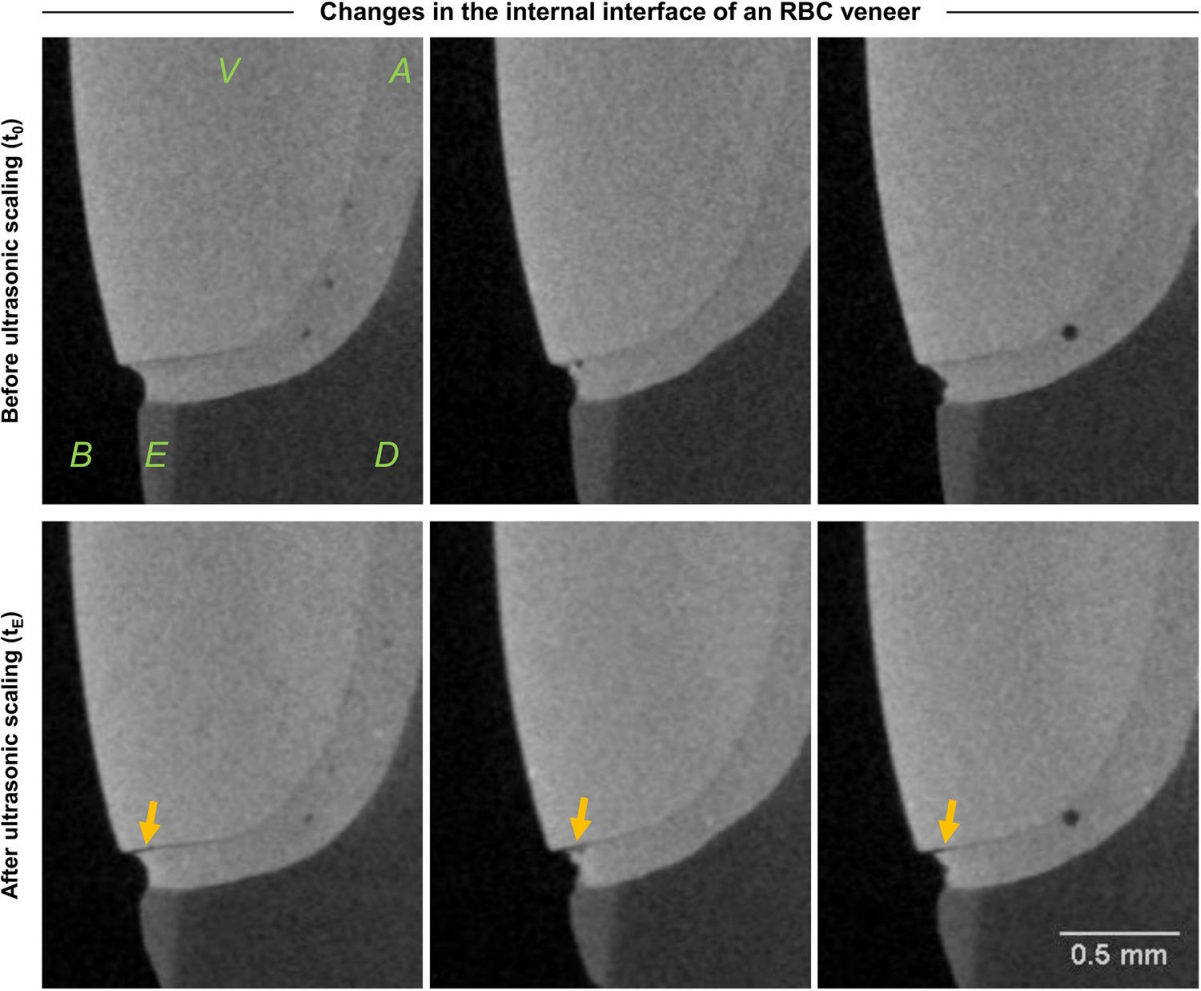
Selected sectional images of a µXCT dataset of a resin-based composite (RBC) veneer with veneer (V), adhesive layer (A), enamel (E), dentin (D), and background (B) before (*t*_*0*_) (upper row) and after (*t*_*E*_) (lower row) ultrasonic scaling with visible alternations/damages in the lower interface area (yellow arrows)

In one of three RBC veneers subjected to ultrasonic scaling, an incipient noncontinuous gap formation in the cervical area of the interface was identified with a depth of up to approximately 0.2 mm (Fig. 4). No definite relation to the observed pores or defects at (*t*_0_) could be established.

### Surface texture

**Fig. 5:**
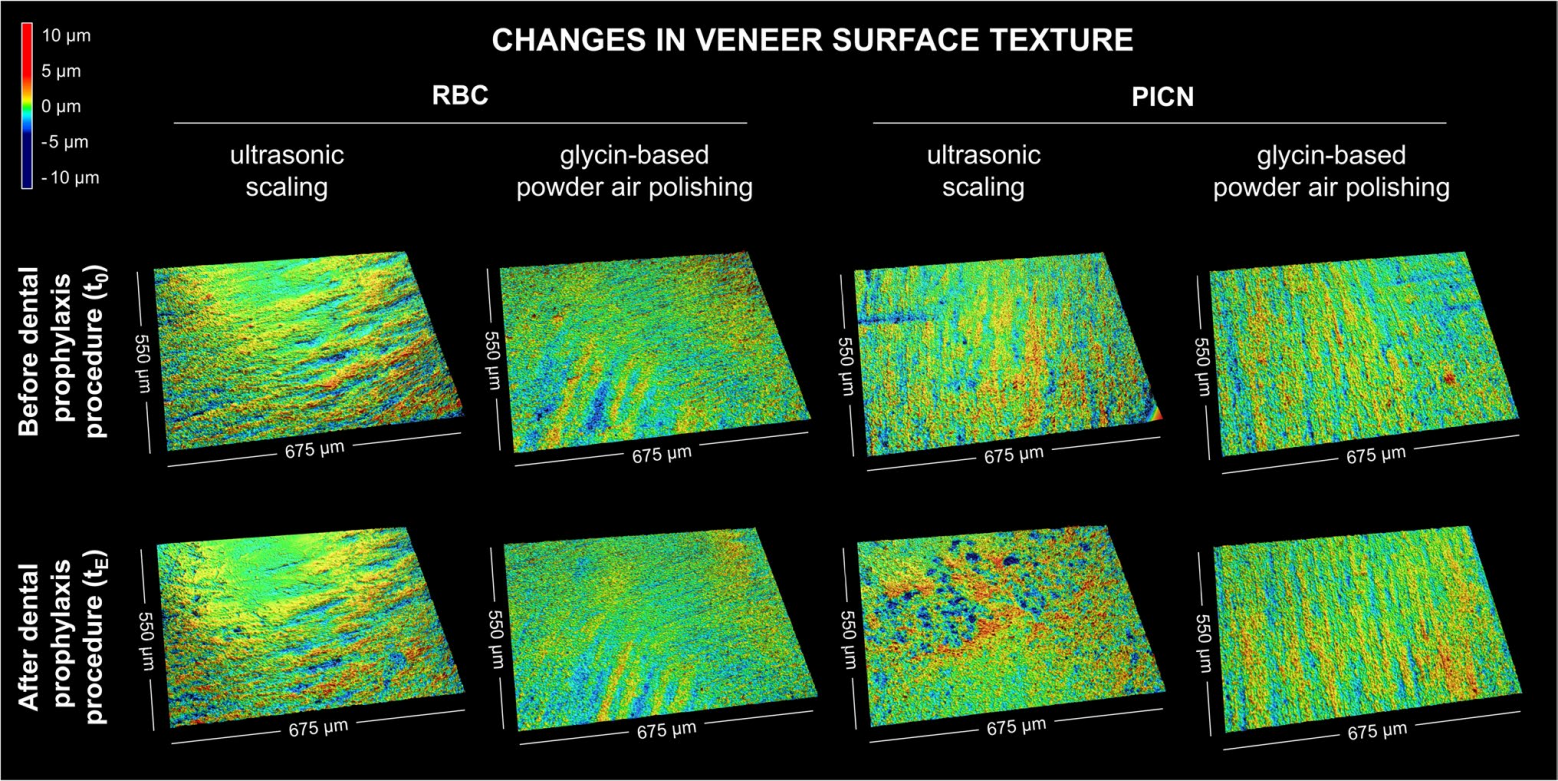
Visual appearance of the surface measurements of resin-based composite (RBC) and polymer-infiltrated ceramic network (PICN) veneers (3D models of S-F surfaces) prior to and after dental prophylaxis procedures by ultrasonic scaling or glycin-based powder air polishing

Ultrasonic scaling particularly produced alterations in the veneer surface (iii) of PICN that were already qualitatively apparent based on the rendered surfaces before and after the prophylaxis procedure (Fig. 5). These modifications can also be retrieved in the analysis of the surface texture parameters of the S-L surface (Table 2). Within the processes from (*t*_0_) to (t_E_), the developed interfacial area ratio (*Sdr*) increased from 8.08 µm (1.18) to 9.59 µm (1.29) *(p* < 0.05). In addition, a decrease in the reduced peak height (*Spk*) from 0.71 µm (0.06) to 0.08 µm (0.06) and a simultaneous increase in the reduced valley depth (*Svk*) from 0.71 µm (0.07) to 0.91 µm (0.16) (*p* < 0.05) were observed from (*t*_0_) to (*t*_*E*_). In contrast to the similar area fractions of peaks and valleys identified at (*t*_0_), the surfaces after ultrasonic scaling yielded fewer peaks and, to a higher proportion, valleys. Due to the strong reduction of the peaks (decreasing *Spk*), a significant increase in the height kurtosis (*Sku*) from 4.04 µm (0.28) to 5.17 µm (0.76) was evident *(p* < 0.01). No significant changes in surface morphology were identified in PICN restorations after air polishing and RBC restorations after both air polishing and scaling.

**Table 2:** Measured surface parameters based on S-L and S-F surface filtering before (*t*_0_) and after (*t*_*E*_) prophylaxis procedure (*SD*, standard deviation) and accelerated aging with associated statistical analysis

	Surface parameter	Resin-based composite						Polymer-infiltrated ceramic network					
		Ultrasonic scaling			Air polishing			Ultrasonic scaling			Air polishing		
		*t*_*0*_	*t*_*E*_	*p value*	*t*_*0*_	*t*_*E*_	*p value*	*t*_*0*_	*t*_*E*_	*p value*	*t*_*0*_	*t*_*E*_	*p value*
S-L surface (roughness)	*Sa/µm*	0.44 (0.12)	0.47 (0.15)	0.643	0.44 (0.10)	0.39 (0.03)	0.127	0.50 (0.04)	0.53 (0.05)	0.189	0.52 (0.08)	0.48 (0.03)	0.168
	*Sq/µm*	0.59 (0.15)	0.64 (0.17)	0.561	-0.93 (0.27)	-0.97 (0.06)	0.639	0.65 (0.05)	0.71 (0.07)	0.060	-0.99 (0.12)	-1.08 (0.27)	0.420
	*Sdr/%*	8.38 (3.77)	10.28 (5.43)	0.462	8.43 (3.78)	5.81 (1.33)	0.110	*8.08 (1.18)*	*9.59 (1.29)*	*0.038*	8.88 (3.21)	6.88 (0.76)	0.127
	*Ssk*	-1.02 (0.25)	-1.19 (0.49)	0.426	-0.93 (0.27)	-0.98 (0.06)	0.639	-0.02 (0.13)	-1.06 (0.25)	0.201	-0.99 (0.12)	-1.08 (0.27)	0.420
	*Sku*	4.81 (0.78)	6.38 (2.96)	0.214	6.95 (4.61)	4.00 (0.72)	0.153	*4.04 (0.28)*	*5.17 (0.76)*	*0.005*	4.09 (0.60)	5.48 (3.56)	0.327
	*Sk/µm*	1.16 (0.34)	1.22 (0.47)	0.781	1.19 (0.20)	1.10 (0.10)	0.167	1.37 (0.13)	1.41 (0.19)	0.689	1.45 (0.20)	1.35 (0.12)	0.306
	*Spk/µm*	0.67 (0.18)	0.73 (024)	0.592	0.66 (0.23)	0.52 (0.05)	0.100	*0.71 (0.06)*	*0.08 (0.06)*	*0.021*	0.72 (0.16)	0.62 (0.05)	0.136
	*Svk/µm*	0.71 (0.17)	0.79 (0.10)	0.321	0.70 (0.25)	0.54 (0.04)	0.130	*0.71 (0.07)*	*0.91 (0.16)*	*0.011*	0.75 (0.16)	0.67 (0.07)	0.274
S-F surface (waviness)	*Sa/µm*	0.51 (0.22)	0.44 (0.11)	0.473	0.41 (0.14)	0.34 (0.09)	0.427	0.41 (0.08)	0.46 (0.10)	0.284	0.44 (0.10)	0.45 (0.07)	0.839
	*Sq/µm*	0.64 (0.35)	0.52 (0.12)	0.411	0.49 (0.16)	0.41 (0.09)	0.385	0.49 (0.10)	0.54 (0.12)	0.390	0.54 (0.14)	0.55 (0.10)	0.840
	*Sdr/%*	0.03 (0.01)	0.04 (0.01)	0.351	0.01 (0.01)	0.01 (0.01)	0.896	0.02 (0.01)	0.03 (0.01)	0.351	0.03 (0.03)	0.03 (0.02)	0.554
	*Ssk*	1.49 (0.34)	1.30 (0.16)	0.206	1.39 (0.50)	1.23 (0.12)	0.516	1.24 (0.18)	1.16 (0.25)	0.499	1.20 (0.39)	1.15 (0.56)	0.843
	*Sku*	2.90 (1.58)	2.22 (0.56)	0.299	4.24 (3.26)	2.58 (0.54)	0.259	2.48 (0.36)	2.33 (0.29)	0.413	2.90 (0.98)	3.01 (1.14)	0.849
	*Sk/µm*	0.82 (0.48)	0.86 (0.38)	0.869	0.75 (0.18)	0.70 (0.14)	0.716	0.80 (0.19)	0.81 (0.22)	0.911	0.85 (0.26)	0.84 (0.25)	0.977
	*Spk/µm*	0.69 (0.92)	0.25 (0.09)	0.251	0.37 (0.22)	0.26 (0.06)	0.481	0.34 (0.13)	0.34 (0.11)	0.938	0.48 (0.31)	0.48 (0.29)	0.997
	*Svk/µm*	0.38 (0.20)	0.38 (0.12)	0.990	0.35 (0.12)	0.42 (0.11)	0.163	0.47 (0.26)	0.53 (0.15)	0.596	0.53 (0.33)	0.50 (0.34)	0.842

Changes in surface esthetics were macroscopically visible from (t_0_) to (*t*_*E*_) for veneers fabricated from both restorative materials as well as for the adjacent tooth surfaces. It was perceived that reflectivity decreased, which coincided with less superficial gloss. The transitional interface region appeared deepened, lighter and more accentuated in all specimens at (*t*_*E*_). Example images are included in the supplemental material (Figures S1, S2).

## Discussion

### Survival rate

In the current in vitro study, the veneer restorations featured a high survival rate of 96.9%: 31/32 veneer restorations fabricated from RBC and PICN, which were both adhesively luted and survived five runs of accelerated aging with thermocycling (5/55 °C; 5000 cycles) followed by prophylaxis procedures. The results of this study suggest an increased survival rate for veneer restorations fabricated from RBCs than described in previous clinical trials, which reported clinical survival rates ranging from 87% after 3 years [43] to 75% after 10 years of clinical service [26]. Currently, clinical data available regarding the performance of veneer restorations fabricated from PICN are not available. In contrast to these clinical studies, which analyzed veneer restorations in anterior teeth, the veneers examined in the current study were fabricated for the restoration of the vestibular surfaces of premolars. This procedure was performed as the investigations required numerous natural teeth, and it was simpler to gather premolars than anterior teeth. Thus, the preparation design had to be slightly adapted to respond to the individual tooth anatomy. In contrast to the clinical trials, no chewing simulation was performed in the present study, and all teeth were prepared under standardized in vitro conditions in a laboratory, which might also serve as an explanation for the different survival rates in the current study. Regarding the thermocycling protocol employed, the temperature settings of 5 °C and 55 °C for analyzing adhesive luting to tooth structures are normatively defined [34]. In contrast to temperature, however, the total number of cycles has not yet been standardized. Despite scientific evidence demonstrating that long-term stresses can be simulated in laboratory approaches, an equivalent to physiological aging has yet to be defined [44]. Although Gale and Darvell [35] proposed a thermocycling regime including exposure to three different temperatures (15/35/45 °C; 10,000 cycles) for the simulation of 1 year of clinical service, numerous studies have employed a simulative model with only two distinct temperature settings as a less complex experimental setup. Thus, the approach followed in the current study responds to the recommendation suggesting that 4 days of thermocycling is a sufficient aging protocol [33]. Despite the differences in the aging processes, multiple authors were able to demonstrate that artificial aging has little to no effect on the mechanical properties of RBCs and PICN [45–47].

Furthermore, at least for ceramic veneers, it has been demonstrated that the most important factors affecting the survival rate of interfacial adhesion are preparation design and veneer thickness [48]. These authors found that a ceramic thickness of at least 0.5 mm and a preparation without exposed dentin are beneficial for interfacial bonding. In the present study, these criteria were partially taken into account for the PICN restorations as to their ceramic content. However, an opposite effect has to be expected for RBC veneers, according to which higher bond strength values can be achieved if they are applied directly to dentin instead of enamel [49, 50]. Regarding the preparation design of resin ceramics, no universal recommendation can be found. In general, manufacturers’ instructions should be followed carefully, while there is more freedom in preparation design due to the material properties of the RBC. Therefore, the thickness of the material can be selected more individually than for ceramics, which feature a strong dependence of their individual resistance on predefined parameters [51, 52].

Among all specimens, deterioration of the interface region was visible, which resulted from a decreasing amount of luting composite in the interface area. This phenomenon occurred without any discrepancies in either prophylaxis procedures or RBC/PICN groups. This thesis is partially supported by the study of Andrei et al. (2015), which demonstrated the negative effect of ultrasonic scaling on the adhesive bond [22].

### Marginal tooth veneer interface

Regarding study hypothesis i), a deterioration of the marginal interface region was visible among all specimens, which resulted from a decreasing amount of luting composite in the interface area. This phenomenon occurred without any discrepancies in either prophylaxis procedures or RBC/PICN groups. This thesis is partially supported by the study of Andrei et al. (2015), which demonstrated the negative effect of ultrasonic scaling on the adhesive bond [22]. A possible roughening of the surface of the luting composite is conceivable due to the decrease in gloss, which could lead to the observed brightening of the veneer in the interface area due to the resulting scattering of light. The high standard deviations of the DiffMean and DiffMax values, which in some cases were more than 50% of the initial value, demonstrate a heterogeneous effect of the prophylactic treatment on different positions of the marginal interface. Moreover, it must be taken into consideration that the individual geometry and orientation of the restored teeth contribute to the high variance of the values, which is why an increase in the number of samples as well as in the number of interface positions to be examined is recommended in future investigations. Due to the resulting high standard deviations, no statistical difference between material or treatment method could be identified. Furthermore, no chemical or phase analysis was performed as part of the study. Any changes in the phase composition or polymer structure in the restorative material or luting composite could not be investigated and thus could not be excluded. Simulation of pH changes on the test specimens was not performed in the current study, yet might be included in further trials, as acids may have an impact on the surface properties of composite resins and ceramics due to chemical stress and demineralization. With regard to this aspect, changes in surface texture as well as hardness should be considered [53]. Measurement as well as control of pH values should be included in further studies to investigate to what extent these results are related to the chemical environment and not to mechanical and thermal stress. However, it should be noted that an analysis of the height profile via the tooth veneer interface provides a reliable indication of the superficial changes in this region. To the authors’ knowledge, this is the first time such an analysis has been performed on restored teeth in the present study.

### Internal tooth veneer interface

Regarding study hypothesis ii), the analysis of the nonvisible interface region with µXCT revealed no differences between both prophylactic treatments; however, gaps in the interface layer were identified in PICN but not in RBC restorations. The interfacial damage observed in the current study could represent a possible reason for the reduced adhesion strength of PICN, which was previously documented by Ustun et al. (2020) [54]. RBC specimens showed damage and cleavage formation in the surface area but no further damage in the nonvisible interface region. A reason for the different results could be the different modulus of elasticity with 18 GPa for RBC and 30 GPa for PICN (as issued by the manufacturers). Thus, the values of RBC are closer to those of dentin, which range between 11–19 GPa and for which better bonding as a result of the similar Young’s modulus has been reported [55]. The different coefficients of thermal expansion of dental composites (14–50 ppm) and ceramics (12 ppm) can be considered as another cause for material-specific gap formation in the internal interface [56]. Accordingly, the coefficient of thermal expansion of PICN is closer to that of enamel and dentin than that of RBCs due to its ceramic content, but lower than that of luting cement, at which the failure of the restoration occurred at the interface with the veneer. Furthermore, it should be noted that gaps or pores can only be detected as long as the spatial distribution is above the resolution limit of the µXCT. Thus, any possible undetected damage in the interface is below a width of 7 µm. Additionally, an influence on the esthetic appearance of the veneer cannot be excluded, which results from the additional refractive index of air inclusions. Thus, further studies in combination with the analysis of optical properties and µXCT are recommended, which has already been used as a promising approach for the analysis of the interface. In this context, the effect of X-ray radiation in µXCT measurements on the performance of the adhesive bond should also be considered [57].

### Surface texture

In terms of study hypothesis iii), a change in the surface texture of PICN at a smaller scale (S-L surface) after ultrasonic scaling was evident. Our data responds to the results of other studies that investigated PICN with different experimental and methodological approaches. Egilmez et al. (2018) also highlighted changes in the surface roughness of PICN after thermocycling [58]. According to them, RBCs exhibit higher mechanical loading capacity—in the form of lower flexural modulus—than PICN. Thus, lower elastic deformation of PICN can lead to surface damage due to prophylaxis procedure and reduce intra-mechanical stress relief within the material during thermocycling. In addition, based on the changes in surface texture in the PICN group under ultrasonic scaling, it is suspected that this prophylactic treatment method causes more stress on the material. In the study by Lee et al. (2019), no significant changes were identified based on the evaluation of *Ra* value only; however, alterations in surface morphology were noted based on the mapped surfaces [19]. The changes in *Sku*, *Sdr*, *Spk*, and *Svk* values identified in the current study underline this issue and suggest that exclusive analysis of the commonly used *Sa* or *Ra* parameters does not adequately describe the surface of dental restorative materials [39]. As previously demonstrated in several studies, the correlation between bacterial adhesion and the surface of dental materials is another aspect that should be considered when changes in surface morphology are identified and interpreted [21, 22, 59]. In particular, *R*_*a*_ values greater than 0.2 µm are considered to increase microbial adhesion [60–62]. In this study, even the initially identified (*t*_0_) *Sa* values exceeded this threshold. Furthermore, in addition to roughness, other factors, such as surface charge, stiffness, and wettability, influence bacterial adhesion, and motility [63]. In the current study, macroscopic analysis of esthetic changes showed a loss of surface gloss in all specimens, suggesting an increase in reflectivity. However, laser microscopy could not demonstrate this assumption. Reasons for this discrepancy might include the small surface area analyzed, which was due to the geometric shape of the sample. In addition, the limited lateral resolution, which was caused by the 20× objective, potentially influenced the investigation of the surface roughness. With regard to this aspect, fundamental studies investigating the influence of various prophylaxis measures on the surface roughness according to ISO 25178 using flat samples are necessary to increase the precision of the measurement with a suitable large aperture of the microscopic objective. In this case, subsequent polishing of the treated veneer areas could restore the prior gloss. A direct comparison with a tooth surface as a reference is recommended in future studies.

## Conclusion

Within the limitations of this study, thermal stress and the application of prophylaxis measures, such as air polishing and ultrasonic scaling, affected the marginal and internal interface region of veneers fabricated from both RBC and PICN to varying extents. Prophylaxis procedures may produce veneer surfaces with increased roughness, especially in PICN after ultrasonic scaling. Regarding the internal interface, PICN showed partial gap formation between the veneer and the luting composite after exposure to both types of prophylaxis and accelerated aging. Given that the survival rate of the veneer restorations was 96.9% in the current study, it is not clear whether the observed changes have a clinical impact. Thus, further studies should be conducted, including studies performed in the context of mechanical aging (e.g., chewing simulation). Optical analysis of changes in color, gloss, or translucency is recommended in specimens with simpler geometry.

## Supplementary Information

Below is the link to the electronic supplementary material.

Supplementary file1 (DOCX 1612 KB)

## Data Availability

The data sets that were generated and analyzed during the current study on which the calculation of the reported mean values and distributions are based are available from the corresponding author on a reasonable request.
