# Supplementary material for: Influence of dental prophylaxis procedures on the tooth veneer interface in resin-based composite and polymer-infiltrated ceramic veneer restorations: an in vitro study
Source: Clin Oral Investig. 2022 Dec 11;27(6):2595–607. doi: 10.1007/s00784-022-04816-z (PMC10264478; doi:10.1007/s00784-022-04816-z)
Supplement: Supplementary file 1 — Supplementary file1 (DOCX 1612 KB) [file 784_2022_4816_MOESM1_ESM.docx]

| **Supplemental Material** |  |
| --- | --- |

Influence of dental prophylaxis procedures on the tooth-veneer interface in resin-based composite and polymer-infiltrated ceramic veneer restorations: An in vitro study

## Materials for preparation

Table S1 Materials used for the preparation

| Material | Manufacturer | LOT |
| --- | --- | --- |
| Chloramine T trihydrate | Sigma-Aldrich, St. Louis, MO, USA | STBF3027V |
| PrepMarker Set 4663 | Komet Dental, Lemgo, Germany | 00005039, |
| Fine diamonds |  | 00106393, 00104700 |
| Extra fine diamonds |  | 00072677 |
| Enamic 2M2 HT C14 | Vita Zahnfabrik, Bad Säckingen, Germany | 80670, 82090,74760 |
| Grandio Blocs A2 (HT) 14L | Voco GmbH, Cuxhaven, Germany | 1831230, 2014240, 2033230 |
| Cylinder pointed bur 20 | Dentsply Sirona, York, PA, USA | M49601 |
| Cylinder pointed bur 12 S |  | M72231 |
| Step bur 20 |  | M53879 |
| Step bur 12 S |  | M37816 |
| IPS Ceramic Etching Gel | Ivoclar Vivadent, Schaan, Liechtenstein | X39271 |
| Panavia V5 Universal A2 / Kit | Kuraray Noritake ,Chiyoda, Japan | #000066 |
| Universal A2 Panavia |  | 290096 |
| Technovit 4000 Syrup 1 | Kulzer GmbH, Hanau, Germany | R010024 |
| Technovit 4000 Syrup 2 |  | R010023 |
| Technovit 4000 Powder |  | R010028 |
| Air-Flow Perio Pulver | EMS dental, Nyon, Switzerland | 1810021, 1909163 |

## Macro photography

Macro photography was performed at (t_0_) and (t_E_) as reported by Fuchs et al. (2020) (OM-D EM-1, Mark II, OLYMPUS, Tokyo, Japan and MP-E 65 mm f/2.8 1-5x, CANON, Tokyo, Japan; AES-MFT COMMLITE adapter) to capture changes in esthetics. All images (n ≈ 90) gathered for a single analysis were recorded under constant alignment with fixed illumination and manual white balance and were automatically stacked to combine the images with out-of-focus areas into one sharp image (Helicon Pro 7.5.4, HeliconSoft, Kharkiv, Ukraine).


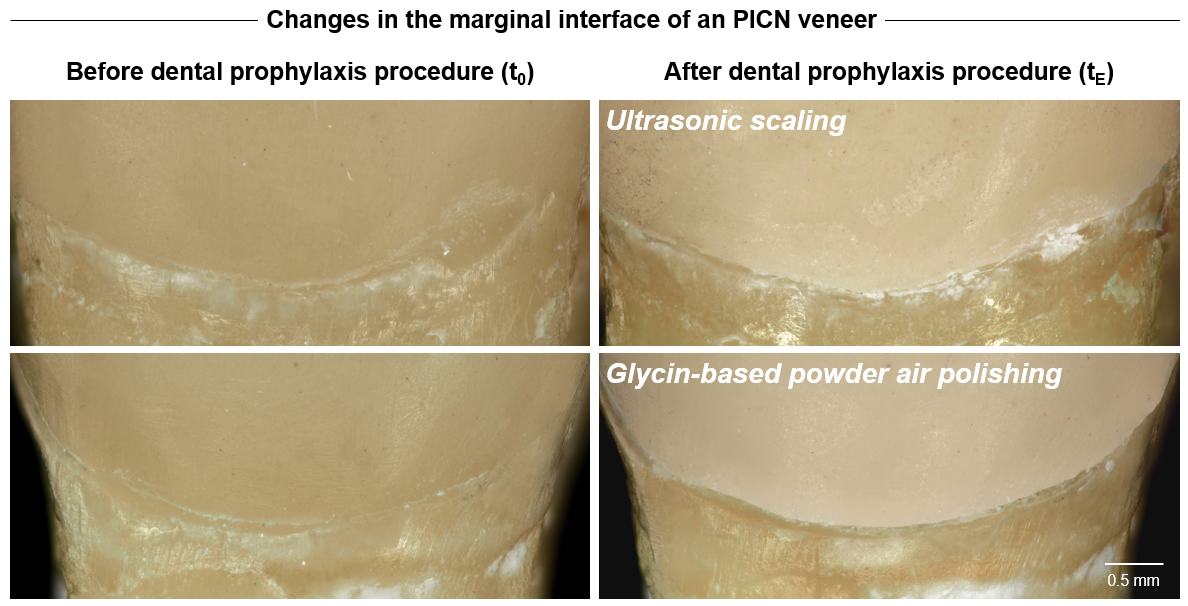


**Figure S1** Comparison of the esthetic appearance of a polymer-infiltrated ceramic network (PICN) veneer prior to (t_0_) and after (t_E_) exposure to prophylaxis procedures.


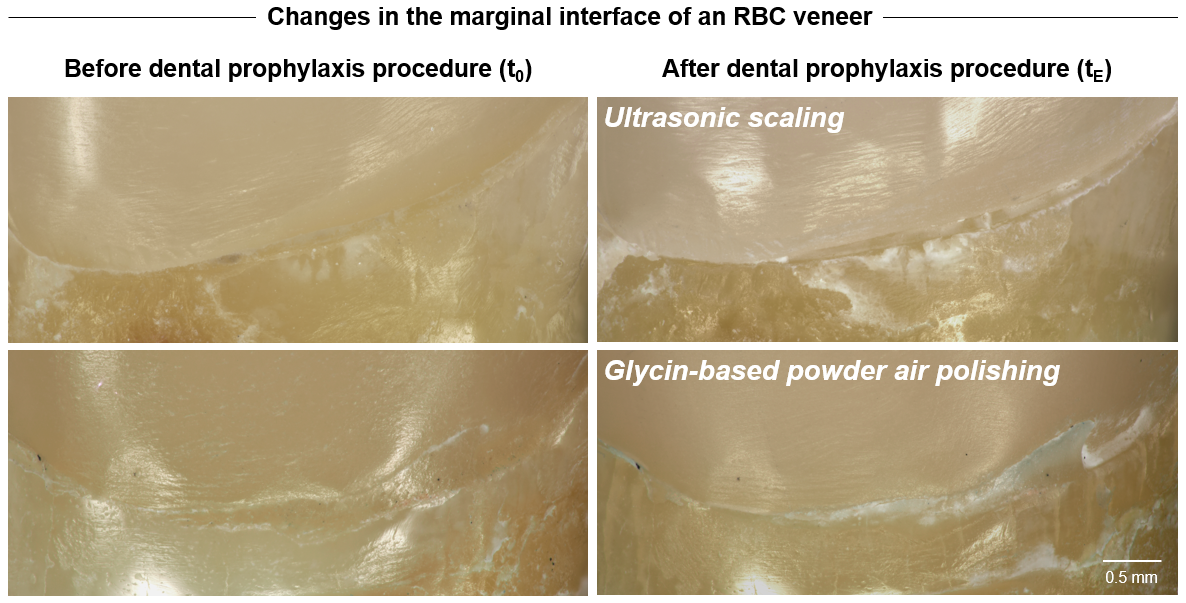


**Figure S2** Comparison of the esthetic appearance of a resin-based composite (RBC) veneer prior to (t_0_) and after (t_E_) exposure to prophylaxis procedures.
